# PENELOPE 1-year follow-up: legacy effect of a short protocol-led LDL-C-lowering strategy in patients after myocardial infarction

**DOI:** 10.1007/s12471-025-01939-2

**Published:** 2025-02-24

**Authors:** Sander van der Brug, Tinka van Trier, Aaram Omar Khader, An-ho Liem, Astrid Schut, Fabrice Martens, Marco Alings, the PENELOPE investigators

**Affiliations:** 1 Department of Cardiology, Amphia Ziekenhuis, Breda, The Netherlands; 2 Department of Cardiology, Amsterdam University Medical Centres, Amsterdam, The Netherlands; 3 Department of Cardiology, Erasmus Medical Centre, Rotterdam, The Netherlands; 4 Workgroup Cardiology, Centres Netherlands, Utrecht, The Netherlands; 5 Department of Cardiology, Franciscus Gasthuis, Rotterdam, The Netherlands; 6 Department of Cardiology, Deventer Ziekenhuis, Deventer, The Netherlands

**Keywords:** Secondary prevention, Low-density lipoprotein cholesterol, Hydroxymethylglutaryl-coenzyme A reductase inhibitors, Ezetimibe, Proprotein convertase subtilisin/kexin type 9 inhibitors, Follow-up study

## Abstract

**Objective:**

Lowering low-density lipoprotein cholesterol (LDL-C) reduces the risk of developing atherosclerotic cardiovascular disease (ASCVD). In the PENELOPE study, a guideline-based, protocol-led LDL-C-lowering strategy was applied in patients after myocardial infarction and resulted in 87% reaching target LDL‑C levels of ≤ 1.8 mmol/l within a median of 45 days. This study evaluated PENELOPE’s legacy effect on LDL‑C levels after 1 year.

**Methods:**

In the PENELOPE study, 999 patients with a myocardial infarction and a history of ASCVD and/or diabetes mellitus were included. If LDL-C > 1.8 mmol/l, lipid-lowering therapy was intensified in three consecutive steps: (1) high-intensity statin (HIST) monotherapy, (2) HIST + ezetimibe, and (3) HIST + ezetimibe + proprotein convertase subtilisin/kexin type 9 inhibitor (PCSK9i). LDL‑C levels were monitored 4–6 weeks after each step. The primary objective of this study was to assess the prevalence of the LDL‑C target level of ≤ 1.8 mmol/l being maintained after 1 year.

**Results:**

Data of 738 patients (74%) were available for 1‑year follow-up. The target LDL‑C level was met in 471 patients (64%). Median LDL‑C levels changed from 1.5 (1.2–1.7) mmol/l immediately after implementation of the protocol-led strategy to 1.6 (1.3–2.0) mmol/l after 1 year. Major treatment regimens were statin (58%), statin + ezetimibe (30%) and PCSK9i + ezetimibe (+ statin) (7%).

**Conclusion:**

After a myocardial infarction, implementation of a protocol-led LDL-C-lowering strategy resulted in 87% of patients attaining the LDL‑C target level of ≤ 1.8 mmol/l within a median of 45 (32–77) days. At 1‑year follow-up, 64% maintained this target level and the median LDL‑C increased by 0.1 mmol/l.

**Supplementary Information:**

The online version of this article (10.1007/s12471-025-01939-2) contains supplementary material, which is available to authorized users.

## What’s new?

- Through a short protocol-led strategy to lower low-density lipoprotein cholesterol (LDL-C) in patients after myocardial infarction, 84% of patients reached the target LDL‑C level using only oral and affordable medication, increasing to 87% with additional use of proprotein convertase subtilisin/kexin type 9 inhibitors.
- At 1‑year follow-up, only 64% of patients maintained this target level. However, the median LDL‑C level increased by only 0.1 mmol/l during this follow-up period.
- This outcome underlines the importance of continued monitoring of lipid panels during the 1‑year follow-up to prevent undertreatment of patients with coronary artery disease.

## Introduction

Patients with atherosclerotic cardiovascular disease (ASCVD) and elevated low-density lipoprotein cholesterol (LDL-C) have a high residual cardiovascular risk [1]. International guidelines recommending strict LDL‑C treatment targets are slowly being adopted in regional multidisciplinary guidelines [2–6]. Despite guideline-based LDL‑C targets, there is a marked disparity with real-world clinical practice [7–9]. These results underscore the challenges in implementing lipid-lowering treatment (LLT) effectively, particularly considering the increasingly stringent LDL‑C guidelines in recent years.

The PENELOPE study assessed the impact of a protocol-led LDL-C-lowering strategy (step 1: high-intensity statin [HIST]; step 2: HIST + ezetimibe; step 3: HIST + ezetimibe + proprotein convertase subtilisin/kexin type 9 inhibitor [PCSK9i]) on achieving guideline-recommended LDL‑C targets in ASCVD patients [10].

Applying the first two steps of this protocol, i.e. with oral LLT only, the target LDL‑C level (≤ 1.8 mmol/l) was met in 84% of patients (intention-to-treat analysis) and 89% (per-protocol analysis), within a median duration of 35 (29–46) days. If PCSK9i was added, the target LDL‑C level was reached in 87 and 95% of patients, respectively [10].

In this follow-up study, we investigated the legacy effect of PENELOPE’s short protocol-led strategy after 1 year. Given that secondary cardiovascular prevention aims to achieve lifetime benefits, these 1‑year follow-up results on LDL-C-lowering strategies are important to assess the need for extended monitoring to optimise long-term outcomes in post-myocardial infarction patients.

## Methods

### Design and study population

The methods in the PENELOPE trial have been described previously [10]. In short, PENELOPE was a multi-centre, prospective, interventional, non-randomised trial. The PENELOPE study enrolled 999 patients aged ≥ 18 years in 23 Dutch centres with a myocardial infarction and a history of atherosclerotic cardiovascular disease and/or diabetes mellitus between January 2019 and August 2020. Main exclusion criteria were a Clinical Frailty Scale score > 3 in patients > 70 years of age, a life expectancy < 1 year, pregnant or lactating women, known intolerance for alirocumab or already using PCSK9i. If LDL‑C was > 1.8 mmol/l, LLT was intensified in three consecutive steps: (1) monotherapy with HIST, (2) HIST + ezetimibe, and (3) HIST + ezetimibe + PCSK9i. LDL‑C levels were monitored 4–6 weeks after each step. All patients gave signed informed consent at inclusion and were invited to sign a re-consent form for the 1‑year follow-up. The trial was sponsored by the Workgroup Cardiology Centres Netherlands (WCN). The WCN was responsible for trial design, site and trial management, and conducted data analyses independently of the funder Sanofi. The ethics committee (Medical Research Ethics Committees United) centrally approved the trial for all participating centres.

### Primary outcomes

The primary outcome of the current analysis was the prevalence of the target LDL‑C level of ≤ 1.8 mmol/l in patients that participated in the PENELOPE study, 1 year after myocardial infarction. Secondary outcomes were median LDL‑C values, LLT regimens and patient-reported therapy adherence.

### Statistical methods

Baseline characteristics are presented using means with standard deviations, medians with interquartile ranges, or percentages for categorical variables. To assess potential selection bias in the 1‑year analysis, we compared the characteristics of patients that were included in the 1‑year follow-up and those who were not. Baseline characteristics (sex, age, medical history) and outcomes after the initial protocol period (LDL‑C target attainment, LDL‑C levels and LLT use) were compared using the chi-square test or Fisher exact test for categorical variables, and the unpaired *t*-test or Mann Whitney U test for continuous variables. The prevalence of LDL‑C target levels and median LDL‑C levels were described for the full cohort. The full 1‑year cohort was the intention-to-treat population, consisting of all patients who signed an informed consent form and for whom lipid panel results after 1 year were available. The per-protocol population was the subgroup without protocol deviations in the treatment phase of the PENELOPE study. In cases where LDL‑C values were not available (*n* = 8) but non-high-density lipoprotein (non-HDL) values were, for the purpose of continuous variables analysis the LDL‑C level was estimated by subtracting 0.8 mmol/l from the non-HDL value. Pre-specified subgroup analyses were performed for age, sex, medical history, oral versus PCSK9i therapy, therapy adherence and therapy changes. Therapy adherence to LLT was patient-reported.

## Results

### Patient characteristics

Data of 738 patients (74%) were available for the 1‑year follow-up visit: 178 patients did not re-sign informed consent, 16 had died, in 38 the cholesterol level had not been measured after 1 year and 29 patients were lost to follow-up. At baseline, i.e. during hospitalisation for myocardial infarction, before protocol initiation, the mean age was 66 (± 9) years, 78% were men, 45% had diabetes mellitus (Tab. 1) and 23% did not use LLT (Tab. 2). Median duration from baseline to the 1‑year follow-up was 384 days (364–435). Among patients who did and those who did not participate in the 1‑year follow-up analysis, there were no significant differences in age, sex and medical history. However, for those who participated, LDL‑C target attainment during the initial study period was higher (90% vs 76%, *p* < 0.001) and median (95% confidence interval) LDL‑C levels were lower (1.5 [1.45–1.5] vs 1.6 [1.51–1.62], *p* < 0.001). The better LDL‑C outcomes among participants were observed concurrently with more intensive LLT, including more frequent use of PCSK9i (5% vs 1.5%, *p* = 0.02) and a higher prevalence of HIST + ezetimibe combination therapy (25% vs 16%, *p* = 0.007). Of the available 738 patients, 31 (4%) did not adhere to the specified protocol during the treatment phase of the PENELOPE study. Therefore, the per-protocol cohort included 707 patients, representing 71% of the initial PENELOPE cohort (Fig. S1, Electronic Supplementary Material).

**Table 1:** Baseline characteristics (before protocol-led care) of all patients with 1‑year follow-up

		Baseline LDL‑C mmol/l, median (Q1–Q3)	Baseline LDL-C ≤ 1.8 mmol/l, *n* (%)
Total (*n*), all patients with 1‑year FU	738	2.1 (1.6–2.8)	283 (38)
*Age, mean (SD)*	67 (± 9)		
– ≤ 70 years, *n* (%)	489 (66)	2.2 (1.7–3.0)	157 (32)
– > 70 years, *n* (%)	249 (34)	1.8 (1.4–2.5)	126 (50)
*Sex, n (%)*			
– Female	163 (22)	2.2 (1.7–3.2)	49 (30)
– Male	575 (78)	2.01 (1.5–2.7)	234 (41)
*History, n (%)*			
– Only DMII	188 (25)	2.39 (1.9–3.2)	43 (23)
– Only ASCVD	406 (55)	2.0 (1.6–2.7)	163 (40)
– ASCVD and DMII	144 (20)	1.7 (1.4–2.5)	77 (53)

If no baseline LDL‑C measurement was available, non-high-density lipoprotein ≤ 2.6 mmol/l was used to determine the proportion of patients on target

*FU* follow-up, *LDL‑C* low-density lipoprotein cholesterol, *DMII* diabetes mellitus type II, *ASCVD* atherosclerotic cardiovascular disease

**Table 2:** Lipid-lowering therapy at baseline (myocardial infarction) and 1‑year follow-up

	Baseline	One-year follow-up
Total, *n*	738	738
*Total statin mono, n (%)*	460 (62)	430 (58)
– HIST, *n* (%)	168 (23)	298 (40)
– Non-HIST, *n* (%)	292 (40)	132 (18)
*Total statin* *+* *ezetimibe, n (%)*	79 (11)	221 (30)
– HIST + ezetimibe, *n* (%)	35 (5)	159 (22)
– Non-HIST + ezetimibe, *n* (%)	44 (6)	62 (8)
Ezetimibe mono, *n* (%)	23 (3)	9 (1)
PCSK9i + ezetimibe (+ statin), *n* (%)	0 (0)	48 (7)
No lipid-lowering therapy, *n* (%)	176 (24)	30 (4)

*Mono* monotherapy, *HIST* high-intensity statin therapy, *Non-HIST* statin therapy, lower or intermediate intensity, *PCSK9i* proprotein convertase subtilisin/kexin type 9 inhibitor

### PENELOPE cohort with 1 year follow-up

**Fig. 1:**
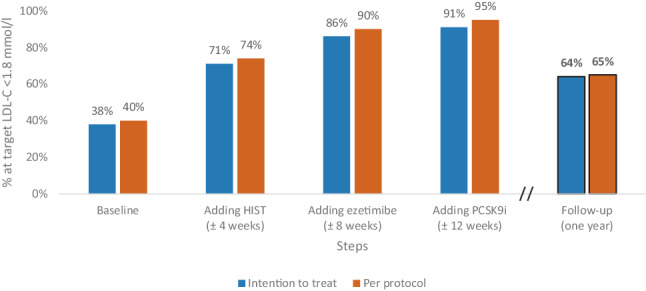
The cumulative effect of adding or adjusting lipid-lowering medication and 1‑year follow-up. The cumulative effect is the percentage of patients treated with lipid-lowering medication who reach the target level (LDL-C ≤ 1.8 mmol/l) in the previous and the corresponding steps. The denominator is 738 patients for the intention-to-treat cohort (all patients with 1‑year follow-up) and 707 for the per-protocol cohort. (*HIST* high-intensity statin, *LDL‑C* low-density lipoprotein cholesterol, *PCSK9i* proprotein convertase subtilisin/kexin type 9 inhibitor)

**Fig. 2:**
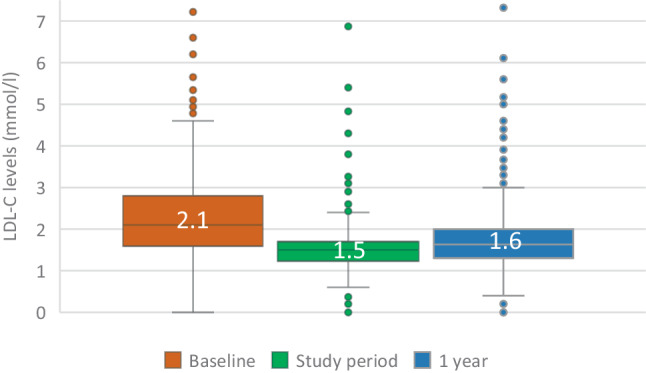
Median low-density lipoprotein cholesterol (*LDL‑C*) over time. The median LDL‑C at baseline, end of PENELOPE treatment phase (study period) and 1‑year follow-up (1 year) in the intention-to-treat population (*n* = 738)

At the end of the intervention phase of the protocol-led LDL-C-lowering strategy, in the 738 patients participating in the 1‑year follow-up, the target LDL‑C level (≤ 1.8 mmol/l) was achieved in 86% (intention-to-treat) and 90% of patients (per-protocol) who were treated solely with oral medication. When including those receiving a PCSK9i this increased to 91 and 95% respectively. The median LDL‑C at the end of the intervention phase was 1.5 (1.2–1.7) mmol/l (Figs. 1 and 2).

**Fig. 3:**
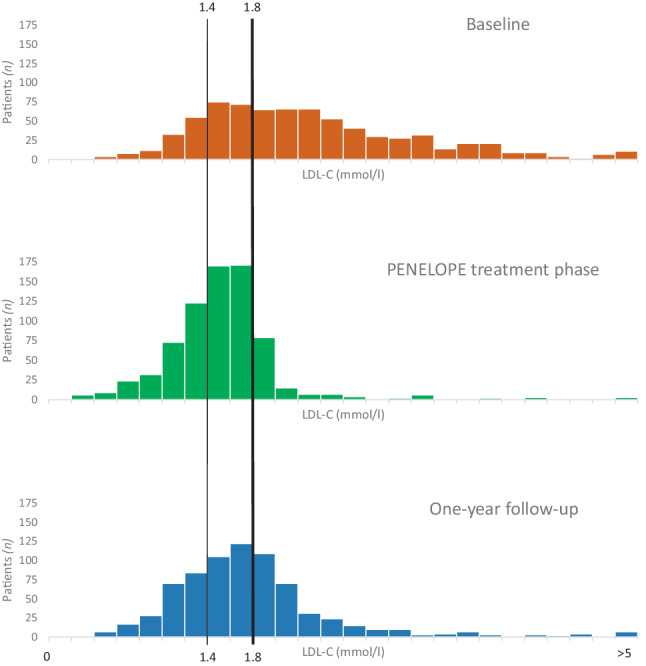
Low-density lipoprotein cholesterol (*LDL‑C*) distribution at baseline, end of study period and 1‑year follow-up. The LDL‑C distribution at baseline, end of PENELOPE treatment phase and 1‑year follow-up in the intention-to-treat cohort (*n* = 738, all patients at 1‑year follow-up)

After 1 year, in 471 of 738 patients (64%) LDL‑C was still ≤ 1.8 mmol/l. The distribution of LDL‑C at baseline and after 1‑year follow-up is demonstrated in Fig. 3. The median LDL‑C level after 1 year was 1.6 (1.3–2.0) mmol/l (Fig. 2).

After 1 year, statin monotherapy was being used by 430 patients (58%), statin + ezetimibe by 221 patients (30%), PCSK9i + ezetimibe (whether in combination with a statin or not) by 48 patients (7%), and ezetimibe monotherapy by only 9 patients (1%). In the main treatment groups, an LDL‑C level ≤ 1.8 mmol/l was maintained in 270 patients (63%) with a median LDL‑C of 1.7 (1.4–2.0) mmol/l, 156 patients (71%) with a median LDL‑C of 1.6 (1.3–1.9) mmol/l and 39 patients (81%) with a median LDL‑C of 1.0 (0.9–1.3) mmol/l, respectively.

Patient-reported therapy adherence was 94%. Thirty patients (4%) were not using LLT; nevertheless, 16 of these patients (53%) reported positive therapy adherence. Patient-reported therapy adherence was seen in 409 (95%) patients on statin monotherapy, in 213 (96%) patients on statin + ezetimibe and in 45 (92%) patients on PCSK9i + ezetimibe (+ statin). The specified treatment groups are demonstrated in Tab. 2.

Between the end of protocol and 1‑year follow-up, a total of 164 patients (22%) were switched to a different LLT regimen. The main reasons for these changes were myalgia (in 65 patients) and LDL‑C levels (in 47 patients). Specified changes are demonstrated in Fig. S2 (Electronic Supplementary Material). Sex, age, medical history and oral versus PCSK9i therapy were not related to target attainment (Fig. S3, Electronic Supplementary Material).

In the per-protocol cohort, 463 of 707 patients (66%) met LDL‑C target levels after 1 year, with a median LDL‑C level of 1.6 (1.3–2.0) mmol/l and a patient-reported therapy adherence rate of 94%.

### Adverse events

Within the 1‑year follow-up period, 71 patients (9%) experienced a serious cardiovascular event, one or a combination of: cardiovascular death (*n* = 10), myocardial infarction (*n* = 26), unstable angina pectoris (*n* = 14), in-stent thrombosis (*n* = 9), revascularisation (*n* = 19), cerebrovascular accident/transient ischaemic attack (*n* = 5), hospitalisation for heart failure (*n* = 13), graft failure after coronary artery bypass graft surgery (*n* = 3) and resuscitated cardiac arrest (*n* = 2). In 41 patients a non-fatal, non-cardiovascular adverse event occurred, and 4 patients died of a non-cardiovascular cause.

### Treatment intolerance

Of the 634 patients without a history of statin intolerance, 65 (10%) developed muscle complaints between the end of the study period and 1‑year follow-up, causing 27 patients (4%) to cease statin therapy, of whom 16 (3%) did not maintain their target LDL‑C level. Six patients (3%) ceased ezetimibe due to side effects between the end of the study period and 1‑year follow-up.

## Discussion

In this follow-up study, we investigated the 1‑year impact of PENELOPE’s initial short intensive protocol-led LDL-C-lowering approach, followed by 1 year of usual care. At the conclusion of the initial interventional phase of the PENELOPE study, LDL-C ≤ 1.8 mmol/l was achieved in 87% of the 999 patients and in 91% of the 738 patients who also participated in the 1‑year follow-up. A favourable leftward shift of the distribution of LDL‑C levels compared to baseline was observed. At 1‑year follow-up, the percentage of patients with an LDL-C ≤ 1.8 mmol/l had decreased to 64%. However, the increase in median LDL‑C was only 0.1 mmol/l (Fig. 2), and the favourable leftward shift of the distribution of LDL‑C levels was largely preserved, indicating that the beneficial effects of a short protocol-led LDL-C-lowering strategy initiated immediately after a myocardial infarction mostly persist at 1‑year follow-up (Fig. 3). Compared to recent observational studies, this incidence of target attainment is relatively high [8, 11], and the median LDL‑C value of 1.6 mmol/l is relatively low [12]. The data from these observational studies align with what is seen in daily practice: far too few patients achieve their long-term LDL target levels. In interventional studies with high-intensity statins (and ezetimibe), short-term results appear to be consistent [13, 14]. Our follow-up study highlights the relevance of including long-term lipid monitoring in secondary prevention implementation strategies.

The decrease in the percentage of patients who maintained the LDL‑C target of ≤ 1.8 mmol/l may have several possible explanations: in 22% of the patients the initial LLT regimen was changed within 1 year and predominantly down-titrated (Fig. S2, Electronic Supplementary Material). Patient self-reported therapy adherence has low sensitivity and specificity [15] and may have overestimated real-life adherence, as other studies demonstrate lower adherence to LLT, especially in the long term [16]. The PENELOPE treatment phase was applied in the first 3 months after a myocardial infarction, the same period in which most patients also participated in cardiac rehabilitation. The combined guidance during the treatment phase of the PENELOPE study, on the one hand, and rehabilitation on the other, may have contributed to better adherence during the first 3 months of the study than during the remaining part of the 1‑year follow-up. This underlines the importance of not switching to less powerful LLT during follow-up and to ensure that patient adherence remains optimal.

Most patients (89%) in the study achieved LDL‑C target levels with oral medication only. Oral LLT is widely available at relatively low cost. Additional, more expensive PCSK9i therapy was necessary in only a limited percentage of patients. Early protocol-led LLT fits the LDL adage ‘the lower, the longer, the better’ [17]. The current data underline the importance of continued monitoring of lipid panels during the 1‑year follow-up to prevent undertreatment of ASCVD patients.

The PENELOPE program shows that current practice falls short in both short-term achievement of the guideline targets (PENELOPE-CTRL) and long-term maintenance of the per-protocol strategy (PENELOPE 1‑year follow-up). These Dutch data are a call for action for all involved in secondary prevention.

### Limitations

Some aspects of this study warrant consideration. Of 999 patients in the original PENELOPE study, 178 (18%) did not provide signed informed consent for the 1‑year follow-up. Patients who waived informed consent had higher LDL‑C levels, suggesting the possibility of healthy participant bias. This bias suggests that the study’s 1‑year findings on the effectiveness of the LDL-C-lowering strategy may be optimistically skewed, as patients with poorer LDL‑C levels were underrepresented. Nonetheless, the absolute differences in LDL‑C levels between participants and non-participants were minimal, at only 0.1 mmol/l.

## Conclusion

After a myocardial infarction, the implementation of protocol-driven LLT for a median duration of 45 (32–77) days led to 87% of patients attaining the LDL‑C target level of ≤ 1.8 mmol/l, with a median of 1.5 mmol/l. During usual care 1 year after the myocardial infarction, maintenance of the target LDL‑C level decreased to 64%, with a median of 1.6 mmol/l. However, the favourable leftward shift in the distribution of LDL‑C which developed during the protocol-led period was maintained.

## Supplementary Information

Figure S1 Flowchart of patients at one year follow-up
Figure S2 Changes of treatment regimen
Figure S3 Boxplot subgroup analyses
Acknowledgements
