# Supplementary material for: PENELOPE 1-year follow-up: legacy effect of a short protocol-led LDL-C-lowering strategy in patients after myocardial infarction
Source: Neth Heart J. 2025 Feb 24;33(4):120–9. doi: 10.1007/s12471-025-01939-2 (PMC11953483; doi:10.1007/s12471-025-01939-2)
Supplement: Supplementary file 1 — Figure S1 Flowchart of patients at one year follow-up Figure S2 Changes of treatment regimen Figure S3 Boxplot subgroup analyses Acknowledgements [file 12471_2025_1939_MOESM1_ESM.docx]

**Content of Supplements**

PENELOPE study one year follow-up: effects of a protocol-led LDL-C lowering strategy in patients post-myocardial infarction.

**Table of content**

Figure S1 Flowchart of patients at one year follow-up 2

Figure S2 Changes of treatment regimen 2

Figure S3 Boxplot subgroup analyses 3

Acknowledgements 3

For definitions, dosing schedule of alirocumab, sample size calculation, protocol, see main paper

Omar Khader, A., van Trier, T., van der Brug, S. et al. Effects of a stepwise, structured LDL-C lowering strategy in patients post-acute coronary syndrome. Neth Heart J (2024).

***
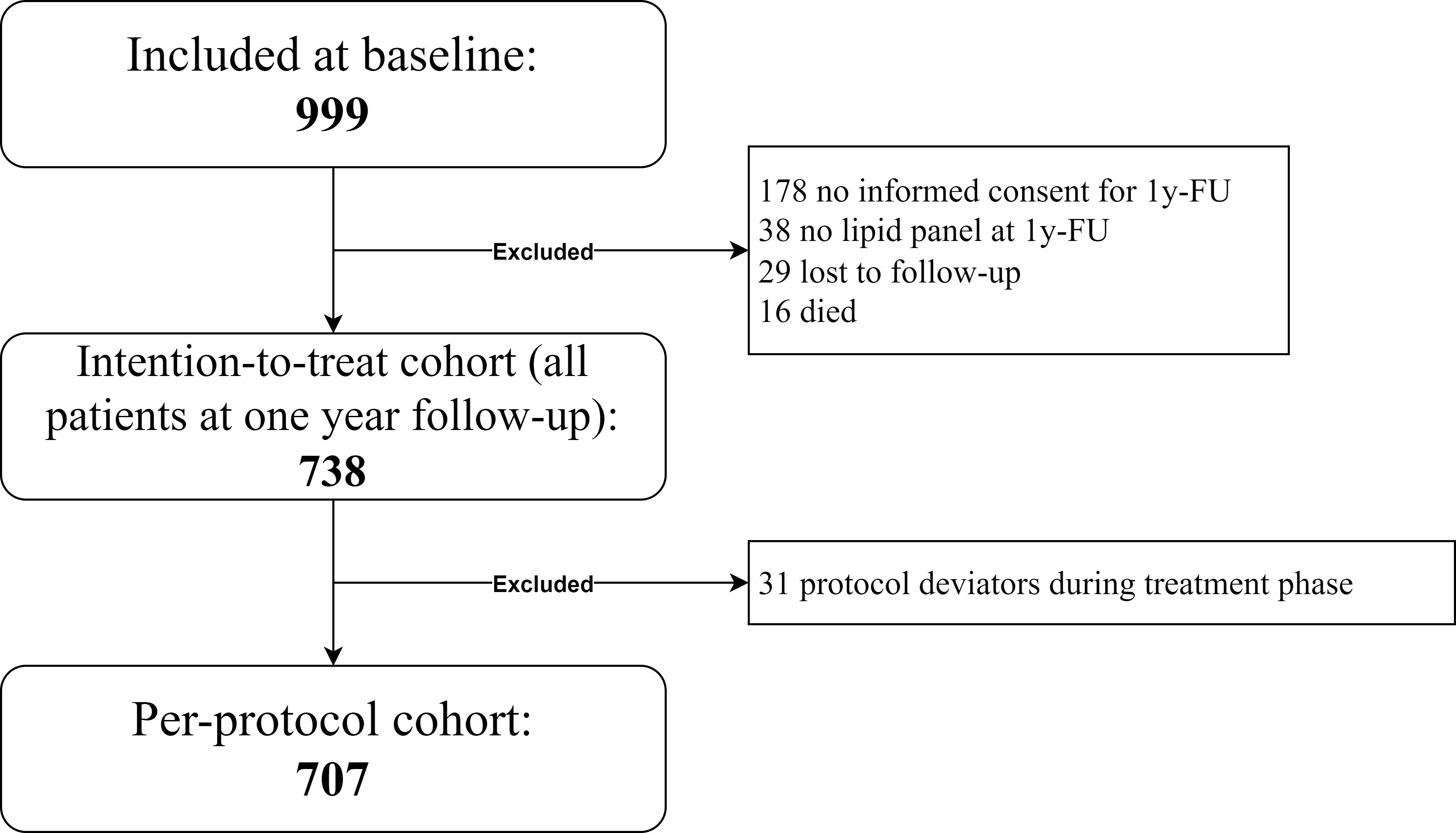
***

**Fig S1 flowchart of patients at one year follow-up.**


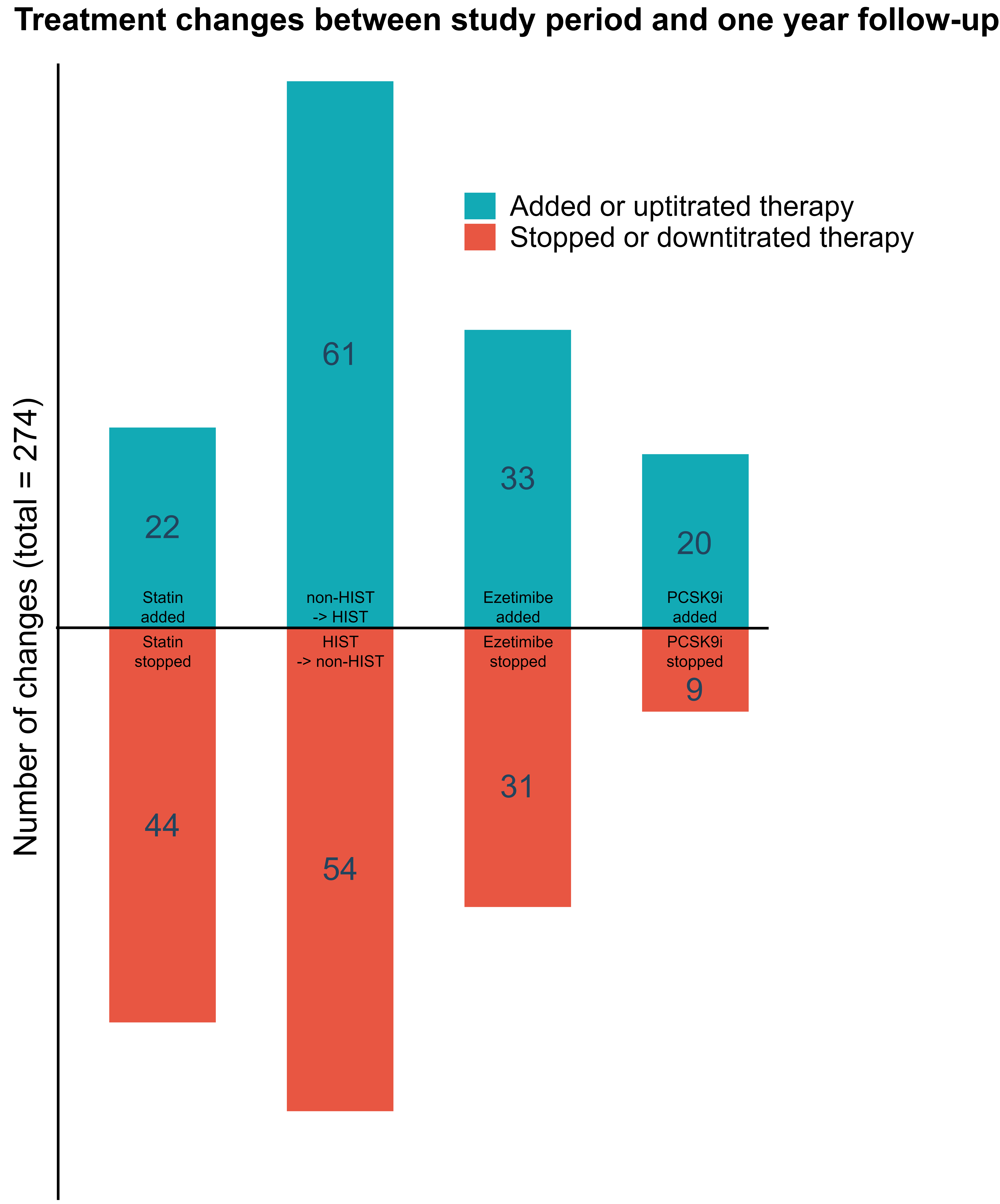


**Fig S2 change of treatment regimen between end of study period and one year follow-up in all patients with one year follow-up (738).** Total of regimen changes: 274; Total of patients with change(s): 164; HIST: high intensity statin therapy; PCSK9i: proprotein convertase subtilisine/kexine type 9 inhibitor.

**Fig S3 subgroup analysis.** Odds ratios with 95% confidence intervals on maintaining target LDL-C levels after one year, per subgroup. Therapy compliant: patient-reported therapy-adherence. LLT: lipid lowering therapy; DM: diabetes mellitus; ASCVD: atherosclerotic cardiovascular disease. PCSK9i: proprotein convertase subtilisine/kexine type 9 inhibitor.

**Acknowledgements**

**PENELOPE Trial Organization**

**Steering Committee Members**

Marco Alings, MD PhD FESC (chair)

F.M.A.C. Martens, MD PhD FESC

A.H. Liem, MD PhD

B.J.B. Hamer, MD

A. Schut, MSc

**Clinical Coordinating Center**

*Dutch Network for Cardiovascular Research, Utrecht, The Netherlands*

Operational Leadership: A. Schut

Project Manager: F. Bindels

Site Managers: S. Hayen and M. van Doorn

Data Manager: M. Atazadah

**Study Sites (number of patients randomized) Investigators**

Jeroen Bosch Hospital, Den Bosch (97): J.W.M. van Eck, D.H. van Dalen

Tergooi Medical Center, Blaricum (71): R.H.J. Peters, G. Nendels

Rijnstate Hospital, Arnhem (67): R. Pisters, F. Peerlings - Terki

IJsselland Hospital, Capelle aan de IJssel (66): D.P.W. Beelen, I.H.G.M. Hendriks

Bravis Hospital, Roosendaal (65): H.G.R. Dorman, T.C. Jacobs

Amstelland Hospital, Amstelveen (59): F.J.F. Broeyer, E. Kooiman

Elisabeth TweeSteden Hospital, Tilburg (57): W.R.M. Hermans, K. Diepen - Dabrowska

Ikazia Hospital, Rotterdam (55): S.K. Zoet- Nugteren, K.A. Visser

Van Weel-Bethesda Hospital, Dirksland (46): C.M. Kievit, G. Oost - Knol

ZiekenhuisGroep Twente, Almelo (41): G.C.M. Linssen, L. Kuipers

Gelderse Vallei Hospital, Ede (40): F.R. den Hartog, D. van Wijk

Slingeland Hospital, Doetinchem (40): D. Schellings †, I. Drost

Amphia Hospital, Breda (38): A.M.W. Alings, S. van Roij

Meander Medical Center, Amersfoort (38): B. Hamer, J.L. van Doorn

Gelre Hospital, Apeldoorn (35): B.E. Groenemeijer, B.C. Pölkerman

Flevo Hospital, Almere (33): A. van der Spank

Spaarne Gasthuis, Haarlem (31): A.F.M. Kuijper, D. Zweers

Admiraal de Ruyter Hospital, Goes (30): H.A.M. van Kesteren, B. Koole

LangeLand Hospital, Zoetermeer (29): P. van der Meer, K. van Aken - Schumacher

Franciscus Gasthuis, Rotterdam (26): P.R. Nierop, I.J. Kort

St.Jansdal Hospital, Harderwijk (26): E. van Beek

D&A Research and Genetics, Sneek (21): W.J.P. Flu, P.M. Meijlis

BovenIJ Hospital, Amsterdam (3): M.L.J.M. van de Wetering, J. Schilder
